# Carpathian Diatomites and Their Applications in Phase-Change Composites

**DOI:** 10.3390/ma18092097

**Published:** 2025-05-02

**Authors:** Agnieszka Pękala, Michał Musiał, Lech Lichołai

**Affiliations:** The Faculty of Civil and Environmental Engineering and Architecture, Rzeszow University of Technology, Poznanska 2a, 35-959 Rzeszow, Poland; apekala@prz.edu.pl (A.P.); lech.licholai@prz.edu.pl (L.L.)

**Keywords:** rock raw materials, phase-change materials, heat accumulators, minerals, circular economy

## Abstract

Based on a review of the existing literature on the use of diatomite and the functioning of phase-change heat accumulators, in this study, we conducted empirical research on the creation of a phase-change composite based on Carpathian diatomite. As part of our mineralogical research, we determined the phase composition of the Carpathian diatomites in this work. Their internal nanostructure was identified. Nanopores create regular systems that, depending on the variety of diatoms, may have sieve, tubular, or “honeycomb” shapes. Diatomites’ internal structure benefits the absorption capacity of phase-change materials (PCM). The obtained calorimetric thermograms of the organic phase-change material and the diatomite compound highlighted an extension of the temperature range in which phase transformation occurs from 4–5 °C (for pure PCM RT28HC) to 15–17 °C for the composites tested with weight proportions of 1:1 and 4:6. In the case of water-rich varieties, the presence of mixed-package minerals, i.e., montmorillonite, with its small size and specific 2:1 package structure, can hinder the penetration and accumulation of PCM. The ability to bind and accumulate heat will be influenced by the size of the diatomite particles or the relative size of the PCM and pores, i.e., structural and textural features.

## 1. Introduction

Diatomites are silica rocks of organogenic origin that are more compact than diatomaceous earth, with varying degrees of lithification. We have encountered varieties with a low degree of lithification and those with a medium degree of lithification. Diatomites are formed via the accumulation of single-celled algae, referred to as diatoms. In diatomites, the opal shells of diatoms are cemented with silica with varying degrees of recrystallisation. Diatomites mainly appear in deeper zones of cold seas with low salinity. They form much less frequently in freshwater lakes, where the biochemical precipitation of silica may occur via diatoms. In modern deep-sea environments, oceanographic research has revealed a connection between diatom muds and centers of volcanic activity, with an abundant inflow of organic substances carried by rivers flowing into the oceans. Diatom silts form a sediment belt around Antarctica, Africa in the Gulf of Alaska, and Japan.

Carpathian diatomites have formed along the eastern and northern shore of the sedimentary basin, the Krosno beds. This is confirmed by the presence of diatomites in the stratigraphic profiles of Romania. The current diatomite distribution can be attributed to the erosion of the youngest Carpathian flysch formations. Carpathian diatomites were identified in South-East Poland by Leszczawka and Krzywe [1,2], and were found to contain tuff inserts and pyroclastic components related to the liparite lava flows of the Inner Carpathians in the period from the Upper Oligocene to the Burdigalian. The formations of Menilite, Krosno, and Strzyżów, together with their basement, reflect the last stage of the synorogenic closing of sedimentary basins in the Polish part of the Flysch Carpathians, accompanied by volcanic activity [3,4,5]. A detailed review of the physical and technological properties of Carpathian diatomites is presented in [6].

### 1.1. Directions of Use of Diatomite Rocks

The latest laboratory research outlines various possible uses and applications of diatomite rocks. The list below addresses only a few of the attempts that have been made to find new uses for diatomites worldwide. Diatomites have been identified as a potential adsorbent for the removal of CO_2_ in the biogas purification process. Studies have shown that diatomite treated with sulfuric acid has the highest adsorption capacity (189.96 mg/g) compared to dry, aqueous and mixed samples, with an optimal acid concentration [7]. It is also a promising material for the synthesis of MFI zeolites. A Brazilian team obtained the desired material typology in the form of aggregated small crystallites with a pore diameter of 0.8 nm. The researchers obtained a product with the characteristics necessary for future adsorption or catalytic tests and the escalation of industrial production [8].

In studies using structurally laminated diatomite monoliths for wastewater treatment, suspensions of diatomite powders were prepared under controlled water freezing. Increasing the permanent load and the freezing rate improved the porous structure of the material. Diatomite monoliths with a pore size of 29.6 µm successfully removed the model dye Rhodamine B from water by adsorption and offered long-term water stability. The monolith dye uptake capacity changed from 1.38 mg/g to 17.04 mg/g for initial dye concentrations between 1.0 and 12.5 mg/L at 298 K and pH = 6, respectively. An analysis of the adsorption data showed that the diatomite monoliths provided efficient mass transfer in the porous laminated scaffold to the adsorption sites and the mass diffusion of dye molecules in water. They were a rate-limiting mechanism for dye removal [9].

Diatomites are also used in nanocomposites. Modified diatomite has been used as a replacement for silica to strengthen and fill three types of rubber. Diatomites have been modified with various modifiers to fill rubbers such as natural rubber, butadiene–styrene rubber, butadiene rubber, nitrile butadiene rubber, ethylene–propylene–diene monomer (EPDM), chloroprene rubber, silicone-methyl-vinyl rubber, fluorine rubber (FKM), and acrylic rubber (ACM). The most suitable diatomite modifier for various rubbers is Si69. Diatomite material achieves better strengthening results than silica, especially in FKM, EPDM, and ACM forms. It can also be dispersed in these matrices; its compatibility is good, and its mechanical properties are excellent [10].

Diatomite is used to form cellulose acetate nanofiber membranes to create electrospun nanofiber membranes with a large specific surface area and a high porosity with fine pores [11]. In studies on geopolymer materials, the effect of replacing metakaolin with diatomite was investigated. The influence of differing ratios of diatomite and metakaolin and the effect of hardening time on the properties of the tested samples were determined, and the mechanical properties of the compressive strength of the geopolymer were tested after curing. The strength of the samples increased concurrently with the amount of diatomite used [12].

In road construction, to improve the performance of conventional asphalt, tests that involve adding modifiers such as diatomite powder and waste engine oil (WEO) have been conducted. Conventional and modified bitumen samples were tested to analyze their penetration, softening point, viscosity, and loss of ignition. The results showed that an increase in WEO content, especially 3% in the modified asphalt, softened the asphalt at a lower softening point and with a higher heating loss. The diatomite powder exhibited the potential to strengthen the bitumen at high temperatures based on the higher viscosity obtained at 165 °C compared to regular bitumen [13]. Research has shown that diatomites have high phase-change material (PCM) absorption and moderate thermal conductivity compared to other minerals used to produce FSPCM. Furthermore, diatomite has excellent thermal stability, and its large inherent surface area grants it the ability to control humidity [14]. Its thermal conductivity ranges from 0.07 to 0.4 W/(m·K) [15].

### 1.2. Use of Mineral Raw Materials with PCM

PCMs are used in construction to increase the thermal capacity of components or building equipment without significantly increasing their mass. They may be directly added to concrete pockets and mortars or used in the form of caps and packages. Mineral raw materials with a porous structure are a good matrix for free-state PCM, allowing PCM to retain its structure during cyclic melting. This is extremely important when using these materials for construction.

This property of diatomites offers at least a partial solution to the problem of separating PCM from the mineral composite matrix when PCM is used without additional coatings. According to [16], it is possible to combine stearic acid and lauric alcohol with purified diatomite to obtain a tight composite. According to [16,17], clay impurities can be removed from diatomites with a HCl solution, and it is possible to increase the hygroscopic properties of diatomite by implementing a concentrated NaCl solution into its structure.

Diatomites were also used in tandem with PCM in [18], where wall claddings containing PCM diatomite and perlite were created. Their results confirmed that this combination successfully reduced the daily temperature amplitude of the modified wall. Furthermore, the tightness of PCM within the composite was proven.

Furthermore, in [19], the authors presented a constant-shape composite containing vermiculite, PCM, and diatomite. It featured spherical microstructures with increased thermal capacity and could be used as an additive in masonry mortar.

Yang et al. [20] used diatomite and an additional nucleating agent to jointly suppress PCM supercooling. The diatomite initially reduced the subcooling of the PCM, and the additional nucleating agent was further reduced to a lower value. An important aspect of the design of new phase-change mineral composites is the selection of a phase-change substance for the adopted mineral matrix. When the structure and texture of diatomite, as well as its physical and chemical properties, are taken into account, it becomes reasonable to use organic PCM in the form of single aliphatic saturated hydrocarbons or their eutectic mixtures. This choice is due to the chemical inertness of most saturated hydrocarbons to minerals from the diatomite group, according to the authors of [21,22,23].

In addition, as demonstrated in [20,24], the main groups of organic phase-change materials and their eutectic mixtures do not show contraindications to the direct impregnation of diatomite pores. Such substances include saturated aliphatic hydrocarbons and their alcohols, acids, esters, and some polymers.

Moreover, organic PCM is characterized by satisfactory melting/solidification enthalpies and are free from the phenomenon of congruence, as in the case of hydrated light metal salts. Another important aspect supporting the use of saturated hydrocarbons as a PCM is the multiple repeatability of phase transformations, which can be achieved without affecting their thermophysical properties and ability to accumulate heat. PCM also exhibits beneficial behavior in response to overheating and undercooling during rapid temperature changes.

Based on a review of existing knowledge in the field regarding the use of diatomite and the functioning of phase-change heat accumulators, the authors decided to conduct empirical research on the creation of a phase-change composite based on Carpathian diatomite. The newly created diatomite phase-change composite will offer increased energy storage capabilities. Diatomite will act as a matrix of materials, offering stability and increased thermal conductivity.

This is the first study to explore the application of Polish Carpathian diatomites in terms of their use with PCM. It should also be noted that Poland has one of the largest deposits of this raw material in Europe, which differs from diatomites or diatomaceous earths from other locations.

This article offers new information in terms of its verification of the potential enthalpy variability during the phase change of the compound of organic PCM and unpurified Carpathian diatomite, which could not be attributed to the amount of PCM used. In this context, the aim of this research is to determine whether contaminating Carpathian diatomites with clay minerals can create internal structures with organic PCM. Additionally, it is important to ascertain whether the structure of clay impurities and PCM will be unfavorable in terms of the heat capacity of the structure (reduction in the enthalpy of melting and solidification).

## 2. Materials and Methods

### 2.1. Materials

- The organic PCM is a mixture of saturated hydrocarbons. The mixture is characterized by a melting/freezing point of 28 °C and a phase-change enthalpy of 190 J/g. PCM RT28HC was produced by Rubitherm GmbH, Berlin, Germany.
- Carpathian diatomite is a porous, brittle rock with a light cream color. It is characterized by a low bulk density in the range of 1.28–1.38 g/cm^3^. Material with a fraction of 0–2 mm was used in the tests. The material for the tests was prepared in the appropriate proportions of 50% PCM and 50% diatomite, and 40% PCM and 60% diatomite. Diatomite rock samples were collected in Krosno, Poland.

### 2.2. Methods

#### 2.2.1. Mineralogical Research

Our laboratory tests covered a wide methodological spectrum. To identify the structural and textural features and the chemical composition of diatomites in the micro-area, we utilized scanning microscopy via an FEI Quanta 200FEG electron microscope (SEM) (FEI Czech Republic (Cernovická Terasa, Brno, Czech Republic) equipped with an X-ray spectrometer (EDX Genesis) and a backscattered electron (BSE) detector. The research preparation required the sputtering of samples with a gold layer about 30–45 nm in thickness. This process was carried out in a vacuum sputterer. Sample imaging was performed at four magnifications of 2 k, 5 k, 20 k, and 50 k times. The electron acceleration voltage was in the range of 10 to 20 kV.

The phase composition of diatomites was analyzed via X-ray diffractometry using a PHILIPS X’Pert diffractometer with a reflection monochromatizer.

The chemical composition of the diatomites was also examined via the atomic absorption spectroscopy (ASA) method using the PHILIPS PU 9100Xi Camera SX-100 spectrophotometer and inductively coupled plasma atomic emission spectroscopy (ICP AES) using the PLASMA 40 spectrometer.

We began by determining the Al_2_O_3_, Fe_2_O_3_, CaO, MgO, Na_2_O, K_2_O, TiO_2_, and MnO content of the prepared solutions. The amount of phosphorus and sodium was converted into oxides according to the requirements of MGiE instruction no. 3/2002. P_2_O_5_ was quantified using an inductively coupled plasma atomic spectrometer (ICP AES). The SiO_2_ content was determined using the gravimetric method. Five samples were tested in total.

#### 2.2.2. Differential Scanning Calorimetry Tests

The thermal properties of the obtained composite were studied by using the differential scanning calorimetry (DSC) method in the MicroCal apparatus. The tests were carried out on composite samples with various proportions of the organic phase-change material and powdered diatomite.

Laboratory samples of the composite were prepared by heating both components of the composite to a temperature of approximately 30 °C and mixing the organic phase-change material and powdered diatomite in weight proportions of 1:1 and 4:6, respectively. The composites were then subjected to 4–6 melting/solidification cycles by cyclic heating and cooling.

DSC tests were performed on samples weighing 10–15 mg. Each test included two cycles of heating and cooling. The sample heating rate was assumed to be 5 °C per minute, with a temperature change ranging from −10 °C to +50 °C. The error in the instrumental temperature measurement was less than 0.2%, while the error in the measurement of the enthalpy value was less than 2%.

## 3. Results and Discussion

### 3.1. Mineralogical and Petrographic Studies of Diatomites

**Figure 1:**
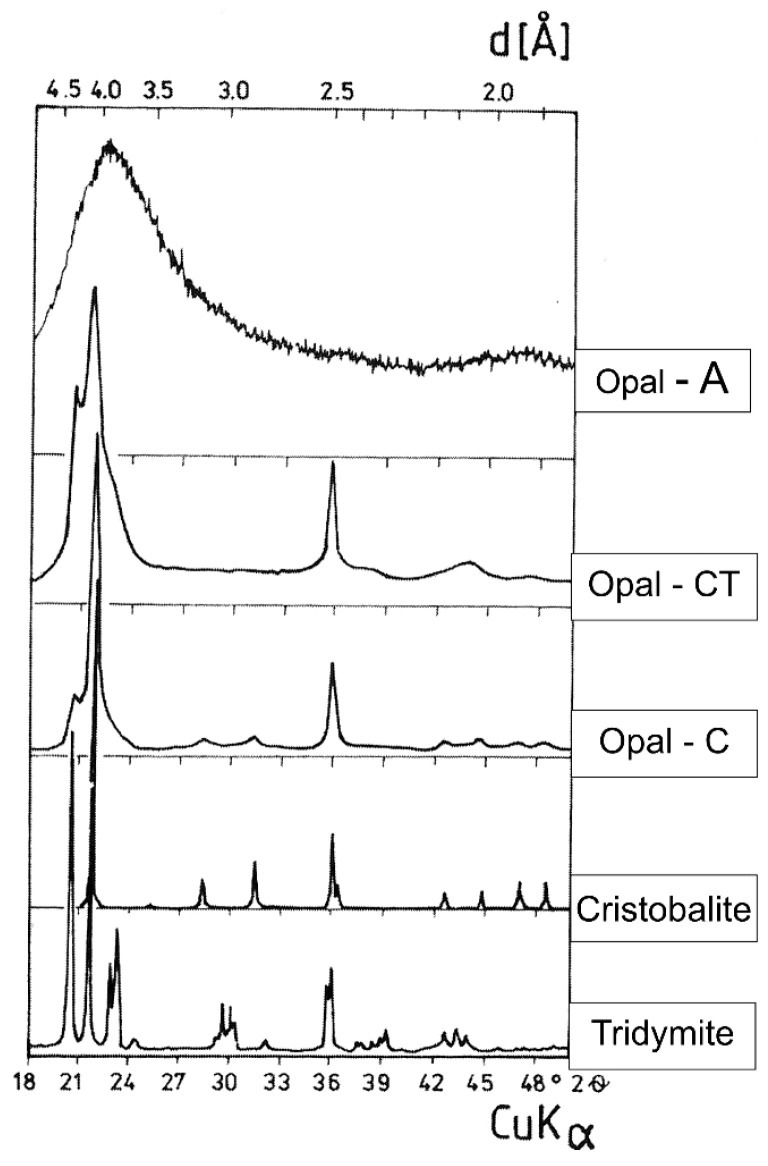
X-ray diffractograms of opal types: A, CT—cristobalite–tridymite, C—cristobalite and, comparatively, tridymite and cristobalite. Explanations of symbols: Copper lamp CuKα, λ = 1.54056 Å; d—interplanar distance.

The Carpathian diatomites used in the experiments exhibited significant petrographic variability and a high degree of mineral contamination with detrital material. Pure varieties of diatomites are very rare; in most instances, they are found as inserts within contaminated varieties. The most common petrographic varieties of Carpathian diatomites are clayey diatomites. The main mineral phase in diatomites is silica, which takes amorphous and crystalline forms. A continuous phase series for silica was found in the diatomites in question (see Figure 1 [25]).

**Figure 2:**
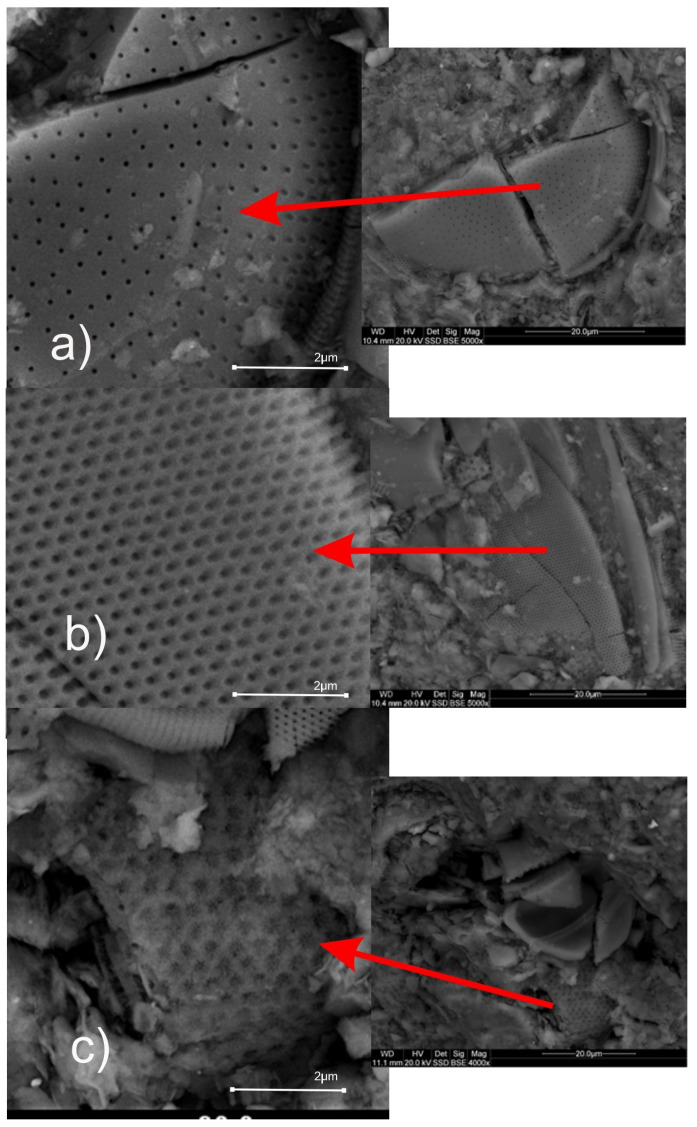
(**a**) Nanostructure of diatoms in diatomites, (**b**) sieve forms of the diatomite structure, and (**c**) honeycomb structure. The images were obtained using an SEM (Scanning Electron Microscope).

The amount of active opal silica is particularly high in the least diagnostic diatomites, ranging from 20 to 40% and sometimes even exceeding 60%. Among the amorphous, siliceous mineral forms, opal types A and CT (cristobalite–tridymite) have been distinguished, and these are mainly used for building bioclasts. The biogenic material in diatomites is mainly represented by diatom shells. Minor sponge needles were identified. Diatomites are made of various types of diatoms, mainly elongated forms of the Pennatae type and oval diatoms. They are characterized by a porous nanostructure and varying shell diameters ranging from 5 to 90 µm (Figure 2).

**Figure 3:**
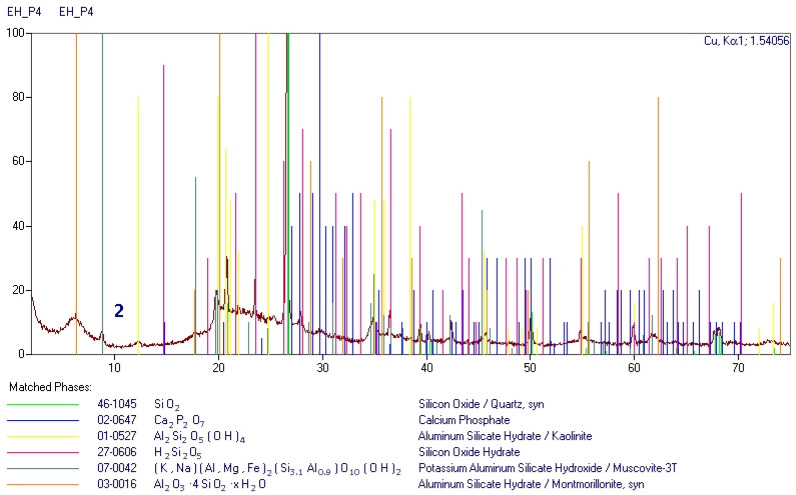
Diffractogram showing phase composition of Carpathian diatomite.

The shells have a layered structure, of which each layer is 50–300 nm thick. The porous nature of diatom shells influences the structural and textural character of the diatomites. Nanopores create regular systems that, depending on the variety of diatom involved, may have sieve-like, tubular, or “honeycomb” shapes (Figure 2). The amorphous form of silica also acts as a binder in diatomites. The opal substance, most likely originating from the dissolution of the diatom shells, cements the detrital material of the rocks. Locally, it is enriched with clay minerals, forming a silica–clay binder, in quantities ranging from 35 to 85%. Clay minerals are mainly represented by illite, the content of which is approximately 15–20% by weight. Montmorillonite and montmorillonite–illite mixed-package minerals were also found (Figure 3), along with trace amounts of nantronite. Silica also occurs in the form of quartz dust, reaching a value of 4–6%. Detrital material is also represented by glauconite, feldspar, mica, pyrite, and goethite. Their content rarely exceeds 2.5%. Furthermore, volcanic glass and bituminous material have been found in the rocks described. The presence of silica or clay minerals in rocks, depending on their percentage and structural and textural characteristics, as determined by diffractometric analyses, has a significant impact on the physical properties of the mixtures. The presence of diatoms composed of amorphous silica has a beneficial effect on the accumulation capacity of PCM; this thesis was confirmed by the authors of [26]. The internal structure of the identified clay minerals within the smectite group may affect their energy storage capabilities. As a result of their packet structure, they possess strong adsorption properties, a large specific surface area, and thermal stability.

Chemical analyses confirmed the siliceous nature of the analyzed rocks (Table 1). The SiO_2_ content ranges between approximately 60 and 90 wt%. There is diversity among fertilized and pure varieties. In the first lithological variety, the silica content is approximately 62%, while in the second, it reaches up to 86% by weight. The content of the remaining ingredients is in the Al_2_O_3_ range of 5 to 10% by weight. The Fe_2_O_3_ content comprises approximately 1% by weight, up to a maximum of 13% by weight.

### 3.2. Experimental Studies

**Figure 4:**
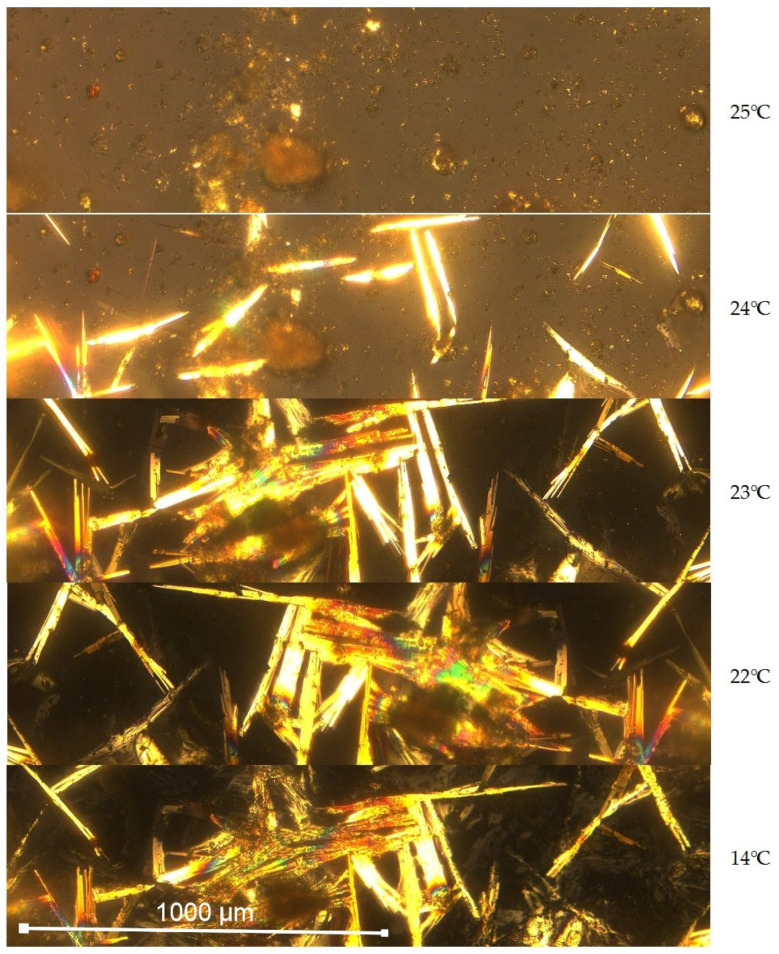
Photographs of the cooled diatomite and PCM composite, taken at 100× magnification.

The results of our tests on the developed composite of diatomite and organic PCM proved the validity of using diatomite as a matrix-binding PCM. The aliphatic mixtures of saturated hydrocarbons saturated the pores and micropores of diatomite, reducing its leakage after melting. During the solidification of the composite, longitudinal (needle-shaped) crystal structures and solid PCM formed near the crystallization grains. The course of the above phenomenon is shown in Figure 4, via microscopic photographs taken at various preparation temperatures.

**Figure 5:**
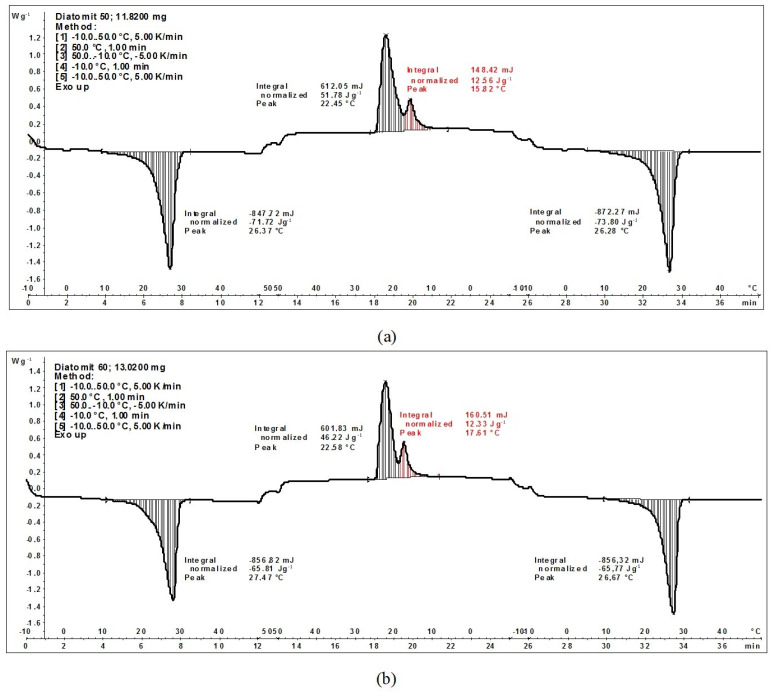
(**a**) Thermogram of a diatomite and PCM in a weight ratio of 1:1; (**b**) Thermogram of a diatomite and PCM in a weight ratio of 6:4.

In turn, the obtained calorimetric thermograms of the organic PCM and diatomite composite showed an extension of the temperature range in which phase transformation occurred from 4–5 °C (for pure PCM RT28HC) to 15–17 °C ± 0.2% for the composites tested with weight proportions of 1:1 and 4:6. Furthermore, two separate peaks were observed during the solidification of the composite, occurring at temperatures of 23 °C ± 0.2% and 16 °C ± 0.2%. The calorimetric thermograms are shown in Figure 5.

Moreover, the Carpathian diatomite, which contains finer mineral fractions, reduced the expected phase transformation enthalpy of the obtained composite. According to stoichiometric predictions, the enthalpy of the melting/solidification of the composite containing 50% diatomite should be 95 J/g ± 2%, but in our experiment, it was approximately 73 J/g ± 2%. A similar trend was observed for the composite containing 60% diatomite. In this case, the expected enthalpy value is 76 J/g ± 2%, compared to the recorded value of 65 J/g ± 2%. This represents a decrease of approximately 24.2–14.5%.

The decrease in the enthalpy value may be due to the interaction of the applied organic PCM with the clay minerals in the impurities. The result of binding the saturated hydrocarbons in PCM into the structure of clay materials is characterized by the occurrence of weak Van der Waals bonds. This is important because the improper or incomplete purification of diatomites from clay minerals can significantly reduce the justification for their use as a matrix for organic PCM.

**Figure 6:**
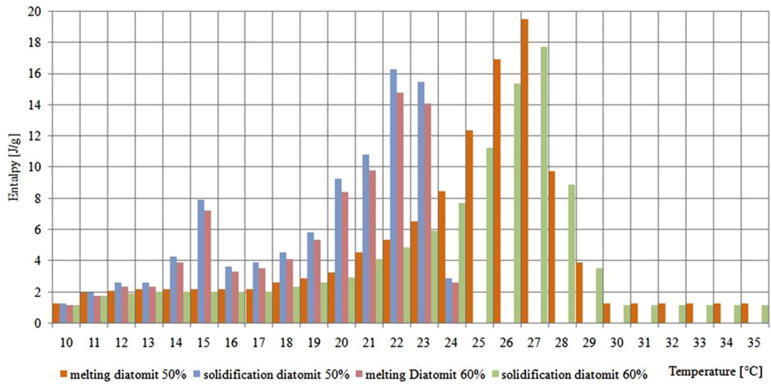
Calorimetric thermogram of a composite of diatomite and PCM RT28 in a weight ratio of 60:40.

The results of the obtained thermograms are summarized in Figure 6, which presents the melting and solidification enthalpy values of the composites tested in a temperature range of 10–35 °C ± 0.2%. In addition to the change in the total value of phase transformation enthalpy between diatomite compounds containing 60% and 50%, we observed a shift in the main peak from approximately 22 °C ± 0.2% to approximately 26 °C ± 0.2%. This proves the formation of new crystal structures of PCM and diatomite, which can create eutectic mixtures which, with the inclusion of the clay fraction of diatomite, may have the potential to increase the enthalpy value. This is an important consideration in the context of the interaction between diatomites, clay impurities, and organic PCM. The insufficient purification of diatomites from clay impurities can cause a double-negative effect. Firstly, it limits the PCM’s ability to penetrate the internal structure of diatomite. Secondly, an unfavorable interaction between PCM and clay impurities further reduces the heat storage capacity of the composite.

## 4. Conclusions

Carpathian diatomites are lithologically and chemically diverse, and the existing varieties are rich in nutrients, though their purity is reduced, with silica constituting almost 90% of the material. Nanopores create regular systems that, depending on the variety of diatoms, may form sieve-like, tubular, or “honeycomb” shapes. The specific nature of the internal structure of diatomites benefits the absorption capacity of PCM.

However, for clay varieties, it is necessary to pay attention to the presence of clay minerals from the mixed-pack group, i.e., montmorillonite. Due to their small size and specific 2:1 package structure, these minerals can fill the porous structures of diatoms, hindering the penetration and accumulation of PCM. The contamination of Carpathian diatomites with very fine grain fractions, such as montmorillonite, makes it difficult for the liquid PCM to penetrate the structure of the diatomite. In future experiments, we plan to separate clay phases from the diatomite rock matrix to improve its accumulation capacity or achieve calcination. Currently, studies show that calcination at 600 °C results in beneficial changes in the internal structure of diatomites that are rich in clay compounds. Thermal treatment induces a general increase in porosity, which is most pronounced in soft varieties. Additionally, clay minerals’ volume changes as a result of the thermal transformation. The internal pores of the silica mineral grains increase because of the initial melting of the silica phases. Diatoms, rocks, and quartz crack under the influence of compressive stresses as a result of changes in the size of growing grains. The organic matter dispersed in diatomites is partially oxidized and removed. At the same time, the structural strength of the tested rocks is improved. The microhardness of the soft and porous varieties of diatomites increased significantly during heating. This was attributed to the presence of clay minerals [27].

The materials’ structural and textural characteristics represent significant advantages in our research, favoring the absorption capacity of aliphatic PCM.

Studies have shown that clay impurities in natural Carpathian diatomites can cause the clogging of their internal pores and may have unfavorable consequences in terms of the enthalpy of phase change, eutectic systems, or mixtures. Systems of mixtures of saturated aliphatic hydrocarbons RT28 HC and diatomite impurities were found to cause a 24.2–14.5% decrease in the enthalpy value.

## Figures and Tables

**Table 1:** Chemical composition of Carpathian diatomites. N—number of samples.

Chemical Component	Content [% by Weight]		
	Minimal	Maximum	Average Value (N = 5)
SiO_2_	62.13	85.71	77.45
Al_2_O_3_	5.7	10.39	8.52
Fe_2_O_3_	1.12	12.95	3.25
MnO	0.007	1.191	0.013
MgO	0.39	0.74	0.63
CaO	0.2	0.4	0.29
Na_2_O	0.18	0.51	0.2
K_2_O	0.89	1.79	1.41
TiO_2_	0.212	0.50	0.26
P_2_O_5_	0.02	0.22	0.02

## Data Availability

The original contributions presented in this study are included in the article. Further inquiries can be directed to the corresponding author.
